# Infectious Diseases, Climate Influences, and Nonstationarity

**DOI:** 10.1371/journal.pmed.0030328

**Published:** 2006-08-15

**Authors:** Bernard Cazelles, Simon Hales

## Abstract

Cazelles and Hales discuss a new study that uses a range of mathematical tools to illustrate a clear relationship between climatic variables and the dynamics of cutaneous leishmaniasis.

Complex dynamic relationships between humans, pathogens, and the environment lead to the emergence of new diseases and the re-emergence of old ones. Due to concern about the impact of increasing global climate variability and change, many recent studies have focused on relationships between infectious disease and climate [1–5].

## Climate and Vector-Borne Diseases

Climate can be an important determinant of vector-borne disease epidemics: geographic and seasonal patterns of infectious disease incidence are often (though not always) driven by climate factors [1–3]. Mosquito-borne diseases, such as malaria, dengue fever, and Ross River virus, typically show strong seasonal and geographic patterns, as do some enteric diseases [4]. These patterns are unsurprising, given the influence of climate on pathogen replication, vector and disease reservoir populations, and human societies. In Sweden, a trend toward milder winters and early spring arrival may be implicated in an increased incidence of tick-borne encephalitis [6]. The recent resurgence of malaria in the East African highlands may be partly explained by increasing temperatures in that region [7]. However, as yet there are relatively few studies showing clear climatic influences on infectious diseases at interannual or longer timescales [5].

The semi-regular El Niño climate cycle, centred on the Pacific Ocean, has an important influence on interannual climate patterns in many parts of the world. This makes El Niño an attractive, albeit imperfect, analogue for the effects of global climate change. In Peru, daily admissions for diarrhoea increased by more than 2-fold during an El Niño event, compared with expected trends based on the previous five years [8]. There is evidence of a relationship between El Niño and the timing of cholera epidemics in Peru and Bangladesh [1,9,10]; of ciguatera in the Pacific islands [11]; of Ross River virus epidemics in Australia [12]; and of dengue and malaria epidemics in several countries [13]. The onset of meningococcal meningitis in Mali is associated with large-scale atmospheric circulation [14].

These studies were performed mostly at country scale, reflecting the availability of data sources and, perhaps, the geographically local effects of El Niño on climate. In part because of this geographic “patchiness” of the epidemiological evidence, the identification of climatic factors in infectious disease dynamics, and the relative importance of the different factors, remains controversial. For example, it has been suggested that climate trends are unlikely to contribute to the timing of dengue epidemics in Thailand [15]. However, recent work has shown a strong but transient association between dengue incidence and El Niño in Thailand [16]. This association may possibly be caused by a “pacemaker-like” effect in which intrinsic disease dynamics interact with climate variations driven by El Niño to propagate travelling waves of infection [16]. This work illustrates the importance of accounting for nonstationarity (see Box 1), transient behaviour that can blur the responses of disease processes to climate forcings.

**Box 1. Nonstationarity**

Nonstationarity occurs in three forms:

- Nonstationarity of the *average*, leading to a trend in the observed time series
- Nonstationarity of the *variance*, including changes in dominant periodic components over time
- Nonstationarity of the *relationships between several observed signals*

Different mathematical tools have been used to account for nonstationarity [9,10,16,17]. Wavelet approaches appear particularly appropriate and have been used for both description of epidemiological time series and quantification of nonstationary relationships between epidemiological and environmental time series [16,17]. As with other statistical analyses, the wavelet approach provides no information about the underlying epidemiological mechanisms. However, wavelet analysis can provide useful clues about the nature of the underlying epidemiological processes that should be incorporated in mathematical models [17]. But the modelling approach must also take into account the nonstationary features of natural systems. In this context, Bayesian approaches such as Kalman filtering or particle filtering seem very promising (e.g., [20]).

## A New Study on Cutaneous Leishmaniasis

In this issue of *PLoS Medicine*, new evidence is presented of a relationship between climate and vector-borne disease. Chaves and Pascual [17] use a range of mathematical tools to illustrate a clear relationship between climatic variables and the dynamics of cutaneous leishmaniasis, a skin infection transmitted by sandflies.

In Costa Rica, cutaneous leishmaniasis displays three-year cycles that coincide with those of El Niño. Chaves and Pascual use this newly demonstrated association to enhance the forecasting ability of their models and to predict the epidemics of leishmaniasis up to one year ahead. Interestingly, El Niño was a better predictor of disease than temperature, possibly because this large-scale index integrates numerous environmental processes and so is a more biologically relevant measure than local temperature. As the authors note, the link between El Niño and epidemics of leishmaniasis might be explained by large-scale climate effects on population susceptibility. Susceptibility, in turn, may be related to lack of specific immunity or poor nutritional status, both of which are plausibly influenced by climate.

Chaves and Pascual [17] have identified a robust relationship between climate and a disease that is nonstationary, with changes over time in average incidence and in cyclic components. The dynamics of cutaneous leishmaniasis evolve coherently with climatic variables including temperature and El Niño indices, demonstrating a strong association between these variables, particularly after 1996. Long-term changes in climate, human demography, and/or social features of human populations have large effects on the dynamics of epidemics as underlined by the analyses of some large datasets on whooping cough and measles [18,19]. Another illuminating example is the transient relationship between cholera prevalence and El Niño oscillations [9]. In Bangladesh, early in the 20th century, cholera and El Niño appeared unrelated, yet a strong association emerged in 1980–2001 [9]. Transient relationships between climate and infectious disease may be caused by interactions between climate and intrinsic disease mechanisms such as temporary immunity [10]. If population susceptibility is low, even large increases in transmission potential due to climate forcing will not result in a large epidemic.

## Public Health Implications

A deeper understanding of infectious disease dynamics is important in order to forecast, and perhaps forestall, the effects of dramatic global social and environmental changes. Conventional statistical methods may fail to reveal a relationship between climate and health when discontinuous associations are present. Because classical methods quantify average associations over the entire dataset, they may not be adequate to decipher long-term but discontinuous relationships between environmental exposures and human health [9,16]. On the other hand, nonstationarity of relationships between climate and disease could signal problems for disease prediction (but see [20]). Unless all important effects are accounted for, dynamic forecast models may prove to have a limited shelf life.
